# Immunological evaluation of patients with Alzheimer's disease based on mitogen-stimulated cytokine productions and mitochondrial DNA indicators

**DOI:** 10.1186/s12888-023-04634-x

**Published:** 2023-03-08

**Authors:** Jiewen Huang, Zhen Song, Beiwen Wei, Qingtian Li, Ping Lin, Hui Li, Ke Dong

**Affiliations:** 1 Department of Laboratory Medicine, College of Health Science and Technology, Ruijin Hospital, Shanghai Jiao Tong University School of Medicine, Shanghai, China; 2 School of Global Health, Chinese Center for Tropical Diseases Research, Shanghai Jiao Tong University School of Medicine, One Health Center, Shanghai Jiao Tong University-The University of Edinburgh, Shanghai, China; 3 Department of Clinical Laboratory, Shanghai Mental Health Center, Shanghai Jiao Tong University School of Medicine, Shanghai, China

**Keywords:** Alzheimer's disease, Mitogen, Peripheral blood mononuclear cell

## Abstract

**Background:**

Based on its objective characteristics, laboratory markers have always been the research direction of clinical diagnosis and assessment of mental disorders including Alzheimer's disease.

**Methods:**

MTT Colorimetric Assay, ELISA, and quantitative PCR were used to investigate the responsiveness of peripheral blood mononuclear cells (PBMCs) to mitogen Lipopolysaccharides (LPS) and Phytohemagglutinin (PHA), PBMCs genomic methylation and hydroxymethylation levels, nuclear DNA and mitochondrial DNA damage, respiratory chain enzyme activities, and circulating cell-free mitochondrial DNA levels were detected in 90 patients with Alzheimer's disease.

**Results:**

In the Alzheimer's disease group, LPS stimulated PBMCs viability, TNF-α secretion, PHA stimulated IL-10 secretion, genomic DNA methylation levels, circulating cell-free mitochondrial DNA copies, citrate synthase activity were reduced compared to the control; while the LPS stimulated PBMCs IL-1α secretion, PHA stimulated IL-1α and IFN-γ secretion, plasma IL-6 and TNF-α, mitochondrial DNA damages were increased compared to the control.

**Conclusions:**

The reactivity of peripheral blood mononuclear cells to mitogens, mitochondrial DNA integrity characteristics, and cell-free mitochondrial DNA copies may be used as candidate laboratory biomarkers to help clinical management of Alzheimer's disease.

## Background

With the development of the social economy and the progress of health care, the aging population and aging-related diseases have attracted more and more attention. Degenerative diseases such as dementia have brought a serious burden to society. Dementia is expected to soar to 74.7 million by 2030 and 131.5 million by 2050. As the most common neurodegenerative dementia, especially in the elderly, Alzheimer's disease (AD) accounts for 50–56% of all dementia cases [1].

Effective early diagnosis and treatment monitoring depend on valuable biomarkers for this neurodegenerative disease [2]. The results of a comprehensive number of clinical studies have shown that T-tau, P-tau, Aβ42, and Neurofilament Light (NFL) in Cerebrospinal Fluid (CSF) should be used in clinical practice and clinical research [3–5]. Updated studies also cover retinal changes, body temperature, and several statistical mapping tools [6–8]. In terms of sample collection convenience and patient compliance, peripheral blood test markers are the first choice for chronic diseases. In general, there is still a lack of early peripheral blood test index for AD.

Inflammation in the nervous system is one of the manifestations of many neuro-related diseases, including AD [9]. Cytokines and their changes are important signs to reveal the existence and outcome of inflammation. The correlations and roles of these inflammatory mediators in the genesis of inflammatory effects have attracted much attention [10]. Here we measured cytokine secretions in plasma and the supernatant of peripheral blood mononuclear cells (PBMCs) stimulated by mitogen to assess their significance in AD.

Mitochondrial DNA integrity and gene expression are also indicators of tissue damage and regeneration. Impaired mitochondrial respiration, particularly cytochrome c oxidase (COX) deficiency, has been observed in peripheral platelets of AD patients [11]. Here we screened genomic methylation and hydroxymethylation levels, nuclear DNA and mitochondrial DNA damage, respiratory chain enzyme activities from PBMCs, circulating cell-free mitochondrial DNA levels in peripheral blood of AD patients.

As a complex disease closely related to immunity, the diagnosis and management of AD have always lacked clear laboratory parameters. We detect PBMC functions, plasma cytokines, genome methylation, and circulating mitochondrial DNA in AD patients, hoping to provide further evidence for the laboratory mission of this disease.

## Materials and methods

### Specimen collection and PBMCs separation, culture, and mitogen stimulation

Ninety AD patients were recruited in this study. They were defined according to the Diagnostic and Statistical Manual of Mental Disorders, 5th edition (DSM-5) (American Psychiatric Association, 2013) and ADAS-cog scale ≥ 25. Another 90 healthy people were recruited from the health screening center without any history of psychiatric disorders. Subjects with current infections, or other inflammatory states, were excluded.

We collected peripheral blood samples in 4 ml EDTA tubes and transported them to the lab within 30 min at room temperature. Plasma was obtained by blood centrifugation at 1500 g for 7 min and kept frozen at -80 °C [12]. PBMCs were isolated using Ficoll-Hypaque density gradient centrifugation. Cell viability was assessed by the Trypan blue dye exclusion assay. PBMCs were suspended in RPMI 1640 medium at a concentration of 2 × 10^5^ living cells per ml, containing 1% L-glutamine and antibiotics (penicillin 100 U/ml-streptomycin 100 μg/ml) with 10% heat-inactivated fetal calf serum, seeded in 12 well cell culture clusters and cultured with 100 ng/ml LPS or 10 μg/ml PHA respectively at 37 °C in a 95% humidified 5% CO_2_ cell culture incubator. Cells and supernatant were collected separately 48 h later, aliquoted, and stored at -80 °C before use.

### MTT Colorimetric Assay

MTT cell proliferation and cytotoxicity assay kits (Beyotime Biotechnology, Shanghai, China) were used in this study for the cell viability assay of PBMCs stimulated by LPS and PHA [13]. Human PBMCs were resuspended to a concentration of 2 × 10^4^ living cells/mL. 100 μL aliquot was added immediately to each well of a 96 well flat bottom microplate in triplicate. LPS or PHA were then added to wells to final concentrations of 100 ng/ml (LPS) and 10 μg/ml (PHA). After 72 h incubation at 37 °C at 5% CO_2_, PBMCs viability was assayed with an MTT kit. The ratio (test/control) of optical density at 570 nm was described as PBMCs-viability.

### ELISA for cytokine concentrations, genome methylation and hydroxymethylation

Supernatants from PBMCs culture systems after incubation with LPS or PHA, and plasma samples were used for cytokine detection. To explore the effects on the balance between immune response and tolerance, cellular and humoral immunity in AD, concentrations of PBMCs cytokines (IL-1α, IL-2, IL-6, IL-10, TNF-α, and IFN-γ) were determined by sandwich enzyme-linked immunosorbent assay using a commercially available kit (Beijing 4A Biotech Co., Ltd. China). All subjects were measured three times on the same day.

PBMCs pellets from peripheral blood were used to detect genome-wide methylation and hydroxymethylation with MethylFlash Methylated DNA Quantification Kit (Epigentek, Farmingdale, NY) and operated according to the instructions.

### Nuclear DNA and mitochondrial DNA damage in PBMCs

DNA damage in unstimulated PBMCs was evaluated with quantitative PCR. DNA was isolated from PBMCs samples using the TIANamp Blood DNA Kit (Tiagen Biotech, Beijing, China). DNA concentration and purity were determined by detecting the absorbance at 260 nm and 280 nm with a NanoDrop ND-2000 spectrophotometer (NanoDrop Technology, Rockland, DE). SYBR Green Real Master Mix Kit (Tiagen Biotech, Beijing, China) was used in 25 μl amplification systems with 10 ng template DNA. Primers for human small and large amplicons for nuclear and mitochondrial DNA were designed from previous studies [14, 15]. For nuclear DNA, primers for small amplicon (86 bp) were: TGC TGT CTC CAT GTT TGA TGT ATC T and TCT CTG CTC CCC ACC TCT AAG T, the primers for large amplicon (13,498 bp) were CGA GTA AGA GAC CAT TGT GGC AG and GCA CTG GCT TAG GAG TTG GAC T. For mitochondrial DNA, the primers for small amplicon (107 bp) were: CAC CCA AGA ACA GGG TTT GT and TGG CCA TGG GTA TGT TGT TA, the primers for large amplicons (8843 bp) were: TCT AAG CCT CCT TAT TCG AGC CGA and TTT CAT CAT GCG GAG ATG TTG GAT GG [16]. The procedure is 95 °C for 10 min, followed by 35 cycles consisting of 95 °C in 20 s and 65 °C for 10 min. The mean of the cycle threshold values (CT) was used for the analysis. Nuclear DNA and mitochondrial DNA damage were expressed as lesions per 10 kb DNA according to Evans’s study [17].

### Respiratory chain enzyme activities in PBMCs

Citrate synthase activity was detected at 412 nm at 25 °C in PBMCs’ whole cell extracts using a citrate synthase assay kit (Abcam). Citrate synthase activity was expressed as μg of citrate produced/min/mL following operating instructions. Cytochrome C oxidase activity was detected at 550 nm at 25 °C in whole cell extracts using a cytochrome C oxidase assay kit (Abcam) [18].

### Circulating cell-free mitochondrial DNA levels

DNA was isolated from thawed plasma samples using the TIANamp Blood DNA Kit (Tiagen Biotech, Beijing, China) according to the manufacturer’s instructions for blood and body fluids protocol. Prior to DNA isolation, plasma samples were centrifuged at 10 000 g for 10 min. Quantitative analysis of cell-free mtDNA was performed using quantitative real time polymerase chain reaction (qPCR). The corresponding number of mitochondrial units was calculated using the following formula according to the previous study [19]:

$$mtDNA units= \left(\frac{\frac{\times gram}{\mu l}DNA}{PCR-frag(bp)\times 660}\right)\times 6.022\times {10}^{23}$$

### Statistical analysis

The demographic and clinical variables of the patient and healthy control groups were analyzed by t-test or Mann–Whitney U test or continuous variables and chi-squared for discrete variables. The statistical software GraphPad 8.3 was used in this study. Data were presented as means ± SD. Differences at *p* < 0.05 were considered to be significant.

## Results

### Demographic data

The demographic data of AD patients and control subjects in this study were shown in Table 1. There was no difference in gender, age, BMI, smoking, and education years between these two groups.

**Table 1:** Demographics of AD patients and control subjects

Parameter	AD (*n* = 90)	Control (*n* = 90)	*P* value
Gender (F/M)	52/38	48/42	0.55
Age (years; means ± SD)	72.3 ± 5.9	73.3 ± 5.6	0.24
BMI (means ± SD)	23.1 ± 3.0	23.9 ± 5.2	0.19
Education years (means ± SD)	12.6 ± 3.1	13.0 ± 3.8	0.38
Smoking/No smoking	32/58	26/64	0.34
Duration of illness (months)	19.2 ± 6.3		
ADAS-cog	21.3 ± 5.0		

### MTT colorimetric assay and cytokine levels in mitogen stimulated PBMCs systems and peripheral plasma

**Fig. 1:**
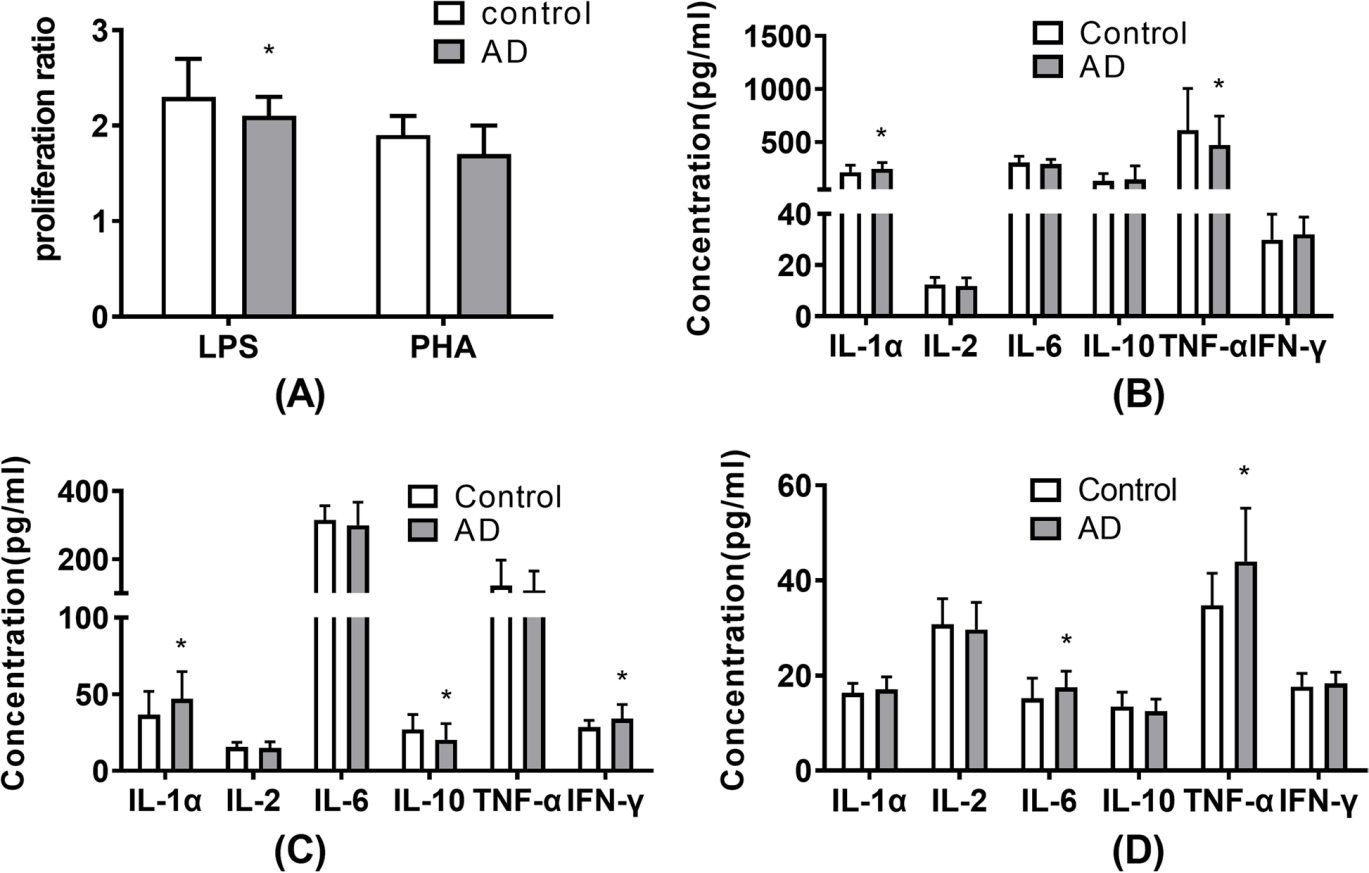
Cell proliferation and levels of cytokines IL-1α, IL-2, IL-6, IL-10, TNF-α, and IFN-γ in mitogen stimulated PBMCs systems and peripheral plasma in 90 AD patients and 90 healthy individuals using MTT colorimetric assay and Double antibody sandwich ELISA. (**A**) LPS stimulated PBMCs showed a lower viability in the AD group. (**B**) LPS stimulated PBMCs produced higher IL-1α and lower TNF-α in the AD group. (**C**) PHA stimulated PBMCs produced higher IL-1α and IFN-γ, lower IL-10 in the AD group. (**D**) Higher IL-6 and TNF-α were detected in peripheral plasma in the AD group. Data presented as means ± SD. Significantly different from controls were signed with * (*P* < 0.05)

MTT test was used to detect the stimulating effects of LPS and PHA on human PBMCs. The viability ratios (test / control) analyzed at 570 nm with an ELISA reader were shown in Fig. 1. The LPS stimulating effects on PBMCs viability in the AD group were significantly lower than those in the control group. For the PHA stimulation assay, there was no significant differences in viability ratios between the AD and control groups.

Double antibody sandwich ELISA kits were used in this study to evaluate various cytokine concentrations in each group. As shown in Fig. 1, when the PBMCs were stimulated by LPS, there were higher IL-1α and lower TNF-α in the AD group than those in the control group, while there was no difference in IL-2, IL-6, IL-10, and IFN-γ between the two groups. In the PHA stimulated groups, higher IL-1α and IFN-γ, lower IL-10 were detected in the AD group than those in the control group. For cytokine production in peripheral plasma, more IL-6 and TNF-α were found in the AD group than in the control group.

### Genome methylation and hydroxymethylation levels in PBMCs

**Fig. 2:**
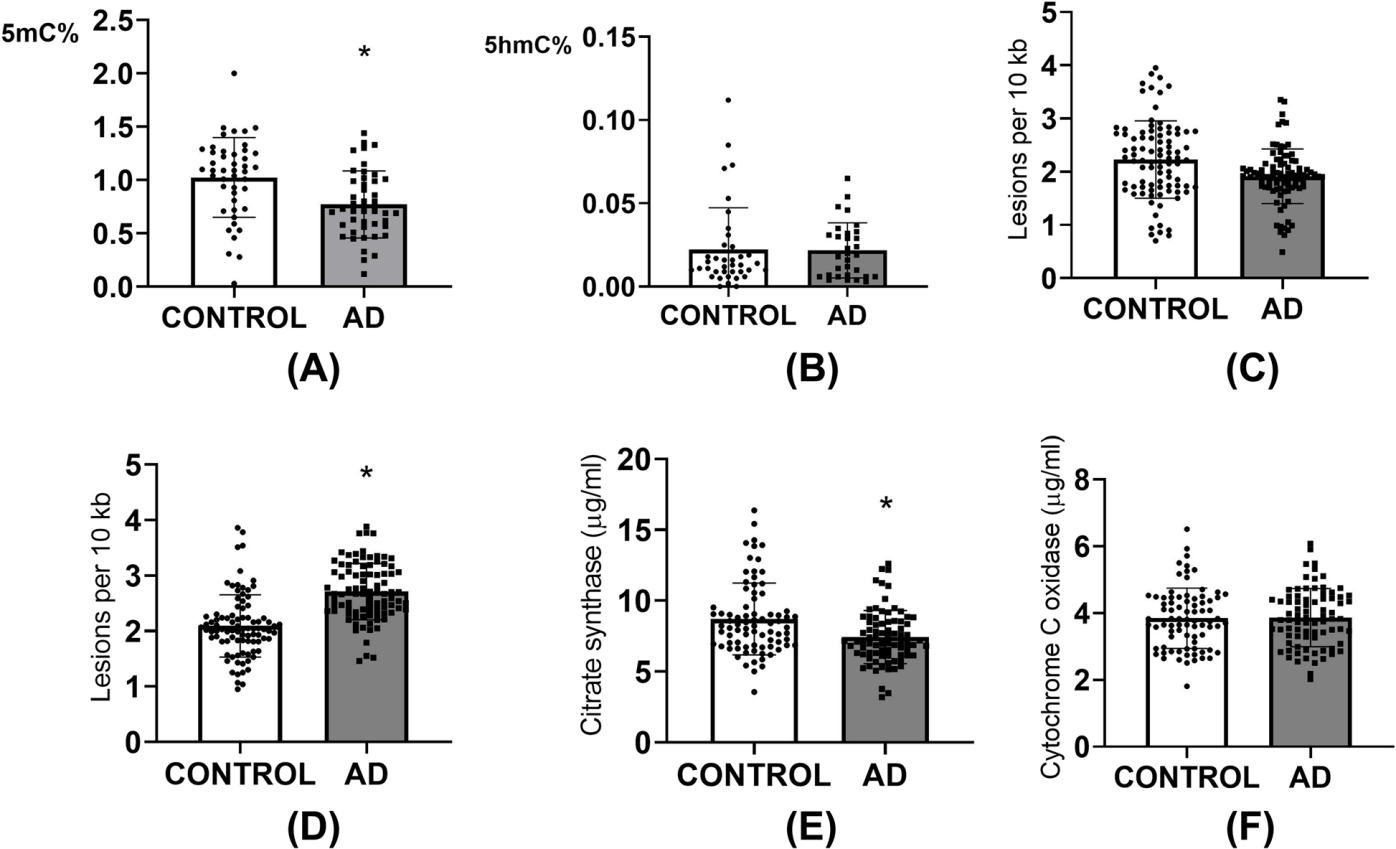
PBMCs genome methylation and hydroxymethylation levels, nuclear DNA damages and mitochondrial DNA damages, Citrate synthase and cytochrome C oxidase activities in PBMCs in 90 AD patients and 90 healthy individuals. Methylated DNA Quantification Kits were used to detect PBMCs genome methylation (**A**) and hydroxymethylation (**B**) levels. Long amplicon real-time quantitative PCR was used to evaluate the nuclear DNA (**C**) and mitochondrial DNA (**D**) integrity in AD patients. Higher mitochondrial DNA damage was found in AD patients. Citrate synthase (**E**) and cytochrome C oxidase (**F**) activities in plasma were detected using qPCR and ELISA kits. Citrate synthase activity was reduced in AD patients. * means significant difference to control (*P* < 0.05)

PBMCs genome methylation and hydroxymethylation levels were detected with a commercial Methylated DNA Quantification Kit. DNA methylation levels in the AD group were lower than those in the control group, while there was no difference in hydroxymethylation levels between the two groups (Fig. 2A, B).

### Nuclear DNA and mitochondrial DNA damage in PBMCs

Large and small PCR products were used to evaluate nuclear DNA and mitochondrial DNA integrity in AD patients, respectively. This method is to estimate the degree of nuclear DNA or mitochondrial DNA damage according to the difference between the product copies after PCR amplification of the long and short amplicons. Compared to the control, mitochondrial DNA damage was significantly higher in AD patients, and there was no difference in nuclear DNA damage between these two groups (Fig. 2C, D).

### Respiratory chain enzyme activities in PBMCs

The activities of citrate synthase and cytochrome C oxidase in PBMCs were measured with homologous kits. Citrate synthase activity was reduced in the AD group, while there was no significant difference in cytochrome C oxidase activity between these two groups (Fig. 2E, F).

### Circulating cell-free mitochondrial DNA levels

**Fig. 3:**
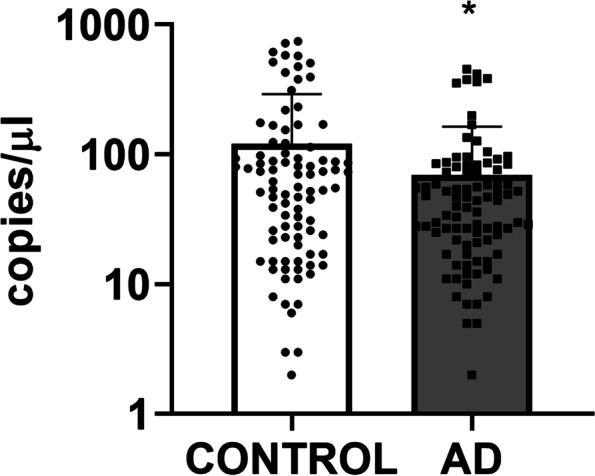
Circulating Mitochondrial DNA copies in the plasma in 90 AD patients and 90 healthy people. Circulating mitochondrial DNA copies were reduced in AD patients. * means significant difference to control (*P* < 0.05)

Circulating mitochondrial DNA was isolated from plasma samples and the quantitative analysis of cell-free mitochondrial DNA was performed using qPCR. Free mitochondrial DNA in the AD group was reduced from those in the control group (Fig. 3).

## Discussion

AD is the most common type of dementia and can seriously affect a person’s ability to carry out daily activities. There are probably many causes or factors, such as age, family history, mental stress, that affect each patient differently. The immune system is a major factor in the occurrence and development of AD [20]. This means that AD is also an inflammation-related disease. However, the exact etiology and determinants of AD outcome are still unclear.

Mitogen LPS and PHA stimulation assay can discover the whole immune response and viability levels of B and T lymphocytes. The viability levels of PBMCs stimulated by LPS and PHA were detected with an MTT test. AD patients showed lower viability in LPS stimulation than the control group, while there was no difference between the PHA groups. LPS stimulation indicates the immune status of B cells, or the body’s humoral immune function [21]. It seems that the humoral immunes of AD patients were injured to a certain extent. Patients with AD have peripheral B cell mediated humoral immune responses to pathological proteins, and the peripheral immunoglobulin repertoire is dysregulated. Changes in peripheral immune system also link Alzheimer's disease with accelerated aging [22]. There is evidence that humoral immunity interacts with Tau protein, although immunoglobulin cannot enter the central nervous system directly [23].

AD is related to the human immune system and the regulations and interactions among those cytokines and immune molecules [24]. In cytokine secretions in LPS stimulation, we obtained higher IL-1α and lower TNF-α in the AD group, while there was no difference in IL-2, IL-6, IL-10, and IFN-γ between the AD and control groups. IL-1 family members can play multiple roles in innate and acquired immunity. The aging process is accompanied by the increase of TNF-α of resting B cells and the inhibition of B cell regeneration [25]. In the PHA stimulated groups, higher IL-1α and IFN-γ, lower IL-10 were detected in the AD group than those in the control group. IL-1 family members are the most potent molecules in the innate immune system [26]. Both LPS and PHA stimulated AD patients showed increased IL-1α in PBMC. As a parameter of damage-associated molecular patterns (DAMPs) [27], the increase of IL-1α can be understood as a reaction of the immune system of AD patients. For cytokine production in peripheral plasma, more IL-6 and TNF-α, and less IL-10 were found in the AD group than in the control group. IL-6 is a rapid response factor related to inflammation, which often requires continuous close monitoring. In animal experiments, it was found that IL-10 decreased and IL-6 increased during aging [28]. It seems a trend towards Th1-type immune response in AD patients based on cytokine results in peripheral plasma [29], although the pattern of cytokine changes in PBMC and peripheral blood is not completely consistent.

DNA methylation and hydroxymethylation are epigenetic modifications that play important roles in regulating gene expression and therefore broad ranges of biological processes and diseases including brain aging and AD [30, 31]. Here we found decreased DNA methylation in the AD group, but there was no difference between the AD and control in DNA hydroxymethylation levels. Lower DNA methylation was found in the hippocampus of AD patients [32]. Hypomethylation is also found in other mental diseases, such as bipolar disorder [33]. The source of samples for psychiatric laboratory testing is a challenging issue. In terms of sample availability and patient compliance, PBMCs have significant benefits in disease monitoring.

DNA damage is a critical pathological cause of AD [34]. Mitochondrial respiration reduces with age, and reduced mitochondrial DNA integrity and mitochondrial function directly promote vascular aging [35]. In this study, we found mitochondrial DNA lesions were significantly increased in AD patients. Mitochondrial DNA lesions appear to be a meaningful parameter when used for AD assessment. We also found that citrate synthase activity was reduced in the AD group. There was lower citrate synthase detected in brain tissues of AD patients in Wei’s study [36]. No difference was detected in cytochrome C oxidase here, while this enzyme was reduced in the brain and platelets of AD patients [18]. Mitochondrial DNA and enzymes are closely related to its functions, such as the production of reactive oxygen species and oxidative phosphorylation. Nerve cells are very sensitive to the damage from reactive oxygen species [37]. Therefore, the damage to mitochondrial DNA and the key enzymes in the oxidative phosphorylation process may be alone or in combination as monitoring markers of mental illness.

As a potential biomarker for AD, circulating cell-free mitochondrial DNA has been analyzed from different body fluids in previous studies [38, 39]. Lower circulating cell-free mitochondrial DNA levels in AD patients were found in this study. In neurological diseases including AD, as a result of alterations in neuronal mitochondrial DNA levels in vulnerable brain regions and reduced mitochondrial DNA release, the decreased amount of CSF mitochondrial DNA is related to cellular dysfunction [40]. Circulating DNA levels can be especially useful as biomarkers for conditions involving organs or structures that are difficult to assess [41]. Changes in mitochondrial DNA copy numbers are implicated in cellular dysfunction with aging [35]. Decreased of circulating mitochondrial copy numbers may mean accelerated aging in AD patients.

There are some limitations to this study. First, we only analyzed patients with mild to moderate AD. In the disease management of AD, we also need to distinguish laboratory indicators of disease severity. Secondly, we did not find any laboratory indicators clearly related to the classification of diseases such as ADAS cog in our existing subjects. As far as the current situation is concerned, laboratory biomarkers related to AD are still in the research stage [42], and disease surveillance of confirmed AD patients may become the next target for biomarker development.

## Conclusions

Increased IL-1α in PBMCs’ responsiveness to mitogen may effectively reflect the injury-related reactions of immune cells, while lower mitochondrial DNA integrity and free mitochondrial DNA levels may explain nerve cell damage and aging. These indicators are expected to become important parameters for Alzheimer's disease surveillance in the future.

## Data Availability

The datasets used and/or analysed during the current study are available from the corresponding author on reasonable request.

## Abbreviations

AD: Alzheimer’s disease
COX: Cytochrome c oxidase
CSF: Cerebrospinal Fluid
DAMPs: Damage-associated molecular patterns
DSM-5: Diagnostic and Statistical Manual of Mental Disorders, fifth edition
LPS: Lipopolysaccharides
NFL: Neurofilament Light
PBMCs: Peripheral blood mononuclear cells
PHA: Phytohemagglutinin
